# Bronchoscopic fibered confocal fluorescence microscopy for longitudinal *in vivo* assessment of pulmonary fungal infections in free-breathing mice

**DOI:** 10.1038/s41598-018-20545-4

**Published:** 2018-02-14

**Authors:** Liesbeth Vanherp, Jennifer Poelmans, Amy Hillen, Kristof Govaerts, Sarah Belderbos, Tinne Buelens, Katrien Lagrou, Uwe Himmelreich, Greetje Vande Velde

**Affiliations:** 1 Biomedical MRI unit/MoSAIC, Department of Imaging and Pathology, KU Leuven, Herestraat 49 O & N1 box 505, 3000 Leuven, Belgium; 2 Laboratory of Clinical Bacteriology and Mycology, Department of Microbiology and Immunology, KU Leuven, Herestraat 49 box 6711, 3000 Leuven, Belgium

## Abstract

Respiratory diseases, such as pulmonary infections, are an important cause of morbidity and mortality worldwide. Preclinical studies often require invasive techniques to evaluate the extent of infection. Fibered confocal fluorescence microscopy (FCFM) is an emerging optical imaging technique that allows for real-time detection of fluorescently labeled cells within live animals, thereby bridging the gap between *in vivo* whole-body imaging methods and traditional histological examinations. Previously, the use of FCFM in preclinical lung research was limited to endpoint observations due to the invasive procedures required to access lungs. Here, we introduce a bronchoscopic FCFM approach that enabled *in vivo* visualization and morphological characterisation of fungal cells within lungs of mice suffering from pulmonary *Aspergillus* or *Cryptococcus* infections. The minimally invasive character of this approach allowed longitudinal monitoring of infection in free-breathing animals, thereby providing both visual and quantitative information on infection progression. Both the sensitivity and specificity of this technique were high during advanced stages of infection, allowing clear distinction between infected and non-infected animals. In conclusion, our study demonstrates the potential of this novel bronchoscopic FCFM approach to study pulmonary diseases, which can lead to novel insights in disease pathogenesis by allowing longitudinal *in vivo* microscopic examinations of the lungs.

## Introduction

Respiratory diseases are a major cause of death and morbidity worldwide, with pulmonary infections resulting in over 3.5 million deaths per year^1^. In particular, the incidence of fungal lung infections has risen over the past few decades due to an increase in the number of immunocompromised patients at risk^2,3^. As many fungi are ubiquitously present in the environment, inhalation of infectious particles is often a first step in the pathogenesis. This can lead to lung infection in susceptible individuals, which can further disseminate to other organs. Pulmonary mycosis may be caused by either moulds or yeasts, with *Aspergillus fumigatus* and pathogenic *Cryptococcus* species respectively, as frequently reported causative pathogens^4,5^.

Preclinical research in animal models of pulmonary diseases is necessary to obtain a better understanding of disease processes and their response to treatment. However, currently applied techniques in infection research such as histopathological examination of tissues require sacrificing the animal and are therefore limited to single time point information. Preclinical *in vivo* imaging methods are increasingly used in research because of their ability to non-invasively follow up disease development in individual animals. Anatomical imaging by use of computed tomography (CT) or magnetic resonance imaging (MRI) has already been used in the field of fungal diseases for the longitudinal monitoring of pulmonary infections^6,7^. On the other hand, whole-body optical imaging methods such as bioluminescence imaging allow for sensitive and specific visualization of genetically engineered pathogens. Both anatomical and optical imaging have already demonstrated their added value in the field of preclinical research^6,8,9^. However, there is a need for alternative methods that allow the detection of labelled pathogens in an *in vivo* setting with a microscopic resolution to bridge the gap between these *in vivo* imaging techniques and standard *ex vivo* histopathology.

Intravital microscopy is an optical imaging technique complementary to whole-body imaging methods which enables *in situ* visualization of cellular processes on a microscopic level based on detection of fluorescent signals^10^. This method creates the opportunity for specific detection of pathogens in the complex biological context of intact tissues and organs. As access to the tissue of interest is a prerequisite for this type of microscopy, different approaches have been developed over the years. The most commonly applied approach involves the implantation of an optical window above the tissue of interest, which can then be combined with a variety of microscopic techniques^11,12^. However, the implantation of such windows is technically challenging and invasive, in particular for lung imaging, and only allows examination of superficial structures. Fibered confocal fluorescence microscopy (FCFM) is an alternative approach in which the microscope objective is replaced by a bundle of optical fibres^10^. The flexibility of these fibre-optic probes facilitates their insertion into the body or target organ, thereby enabling the imaging of deeper tissues. Previously, Morisse *et al*. have demonstrated the use of FCFM for *in situ* imaging of pulmonary aspergillosis in rats^13,14^. Due to the dimensions of the fibre-optic probe used in this study, a bilateral thoracotomy was needed in order to gain access to the infected lung tissue, thereby precluding its use for longitudinal studies.

Recently, a variety of FCFM probes with different characteristics, such as a small (<1 mm) or large (>1 mm) tip diameter, have opened possibilities for less invasive approaches for FCFM imaging^10^. By using fibre-optic probes with small tip diameters, endoscopic insertion through natural openings like the large airways of the animal becomes feasible and allows studying pulmonary diseases. So far, such bronchoscopic approaches have mainly been limited to larger animal models such as pigs or rabbits^15,16^. However, the development of a similar bronchoscopic procedure in mice would be of primary interest to research given the large availability of established mouse models and immunological resources.

Previous work has already demonstrated that a bronchoscopic approach in mice is attainable, albeit only in ventilated animals^17,18^. In this work, we investigated the feasibility of performing repeated FCFM imaging on the lungs of living, free-breathing mice by using a bronchoscopic approach. In addition, we have assessed the use of this approach for longitudinal follow-up of pulmonary fungal infections in mouse models of *Aspergillus* and *Cryptococcus* infection. Furthermore, the potential of FCFM to differentiate between causative pathogens with distinct morphological characteristics was investigated.

## Results

### FCFM allows for the *in vitro* visualization of GFP-expressing fungal cells

**Figure 1:**
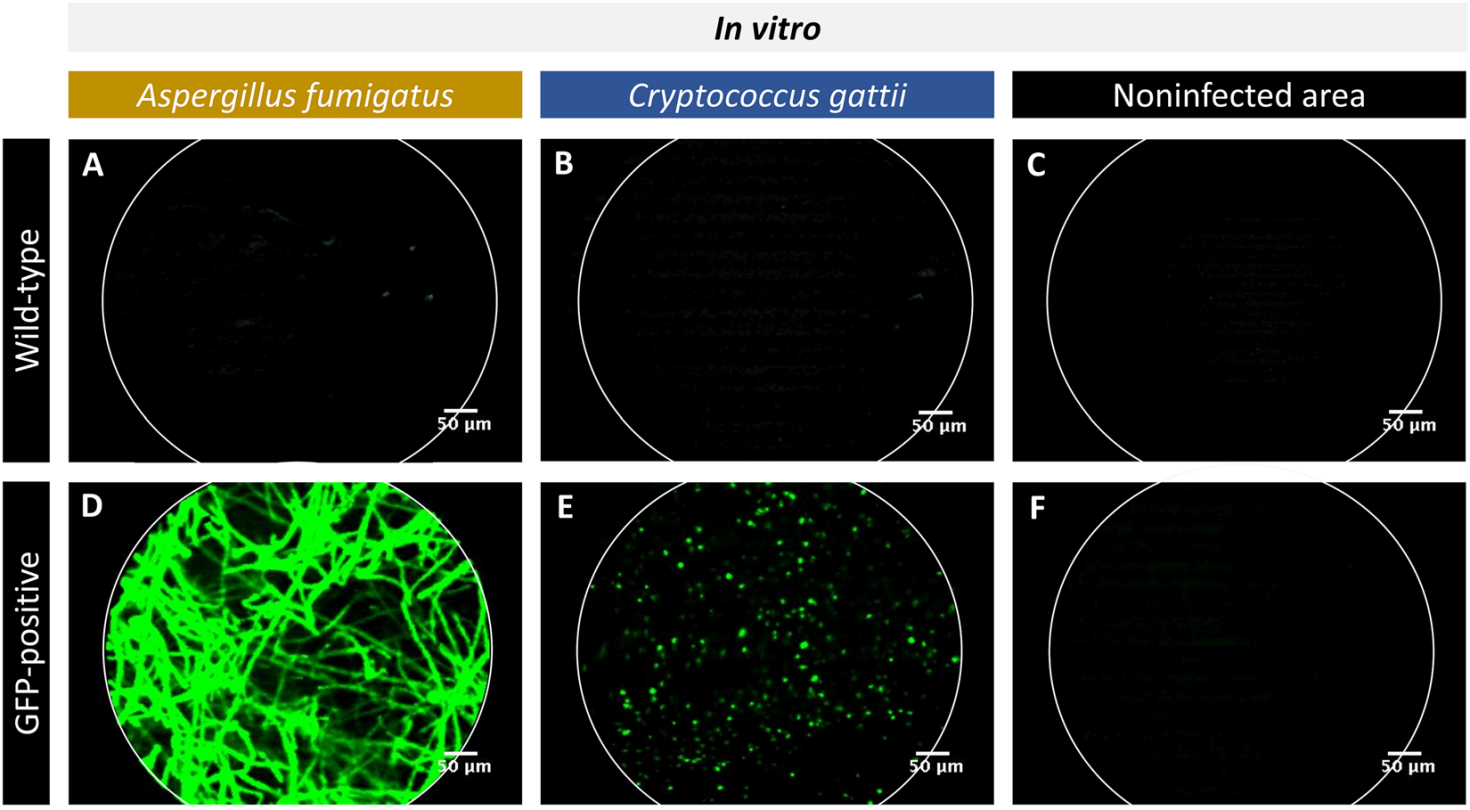
*In vitro* FCFM of different fungal species. The images were acquired by making direct contact between the fibre-optic probe (S-1500) and the fungal colonies grown on solid agar. (**A**–**C**) Representative FCFM images of a WT *A*. *fumigatus* colony, a WT *C*. *gattii* colony and a non-infected region on the agar showing the absence of background signals. (**D**–**F**) Representative FCFM images of a GFP-expressing *A*. *fumigatus* colony, a GFP-expressing *C*. *gattii* colony and a non-infected region on the agar showing the presence of fungal hyphae (*Aspergillus*), cells (*C*. *gattii*) or the lack of background signals, respectively.

To investigate the potential of FCFM to visualize individual fungal cells by using a fibre-optic probe, wild type (WT) and green fluorescent protein (GFP)-expressing *A*. *fumigatus* or C. *gattii* colonies were grown on solid Sabouraud agar and imaged by using the S-1500 probe (tip diameter 1.5 mm). For both fungal species, no signal could be detected when making contact to WT colonies (Fig. 1A,B) or when imaging non-infected agar (Fig. 1C,F). On the other hand, intense fluorescent signals were detected upon direct contact between the densely packed GFP-expressing fungi and the fibre-optic probe (Fig. 1D,E). The images confirmed that *A*. *fumigatus* colonies were composed of densely packed hyphal structures (Fig. 1D), whereas *C*. *gattii* colonies contained a large number of round cells (Fig. 1E).

### The transthoracic FCFM approach enables *ex vivo* imaging of superficially located fungal lesions by using an optical probe with a large tip diameter

**Figure 2:**
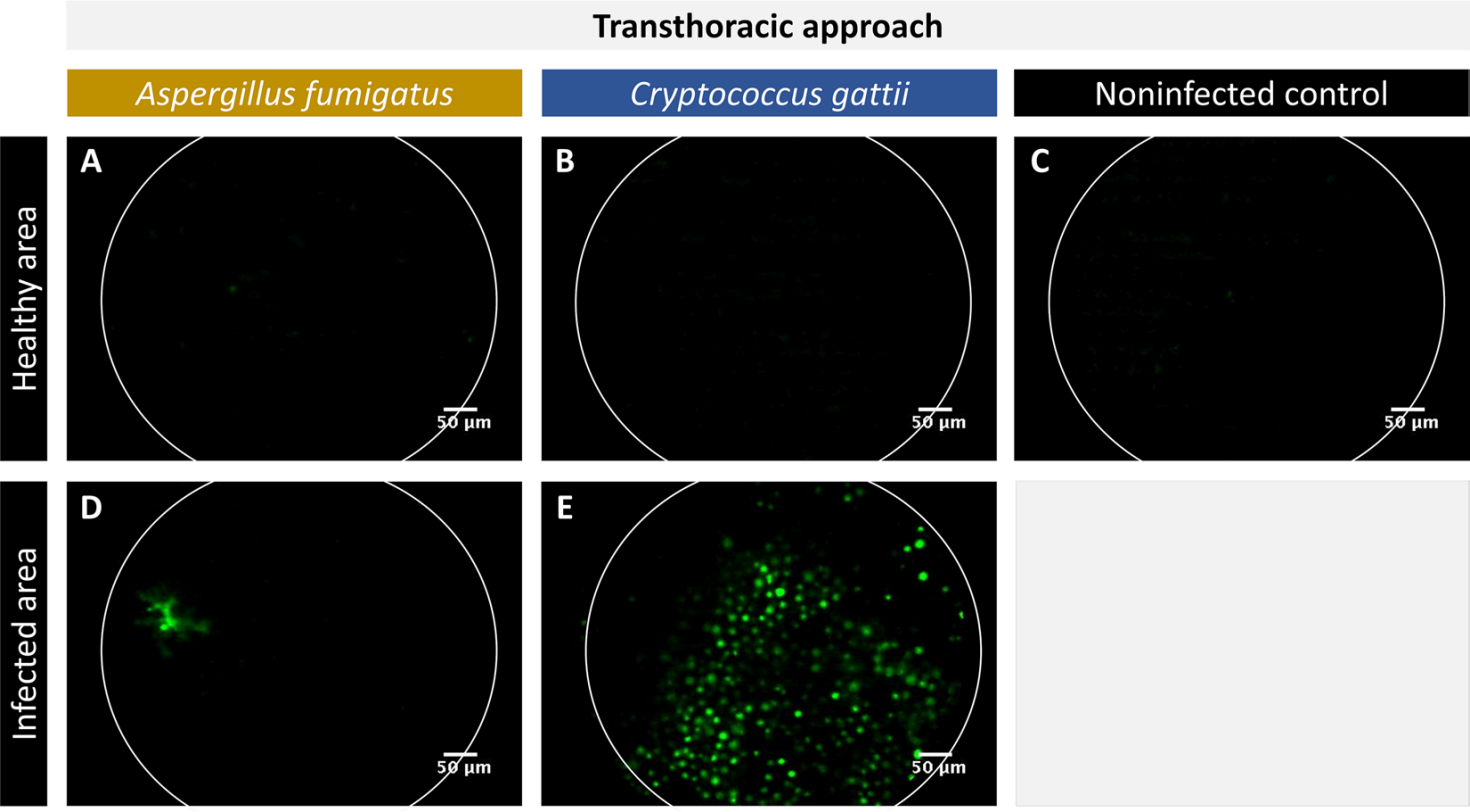
*In situ*, transthoracic FCFM of lung infections. The images were acquired by placing the fibre-optic probe (S-1500) into direct contact with the lung surface after performing a bilateral thoracotomy. (**A**–**C**) Representative FCFM images of a healthy appearing area of an *A*. *fumigatus* (GFP-expressing) infected lung, a *C*. *gattii* (GFP-expressing) infected lung and a non-infected control lung. (**D**,**E**) Representative FCFM images of an infected area of an *A*. *fumigatus* infected lung and a *C*. *gattii* infected lung, showing the presence of fungal cells.

Next, we have explored the use of FCFM for visualizing pulmonary fungal infections in mice. Although the fibre-optic probes are designed for endoscopic use, the S-1500 probe (tip diameter 1.5 mm) was found too large for endoscopic insertion in mouse lungs. Therefore, a bilateral thoracotomy was performed to allow *ex vivo* FCFM imaging of the lungs by placing the S-1500 probe into direct contact with the lung surface. In infected lungs, no signals could be detected when placing the probe against a healthy area containing no GFP-expressing *A*. *fumigatus* or *C*. *gattii* (Fig. 2A,B). Similar results were obtained when imaging a healthy, non-infected control lung (Fig. 2C). In contrast, signals were observed when making contact with a lung area infected with GFP-expressing *Aspergillus* (Fig. 2D). Numerous round GFP-expressing cells were observed when placing the probe against the surface of a nodular lesion found within GFP-expressing *C*. *gattii* infected lungs (Fig. 2E), confirming the presence of a relatively large number of cryptococcal cells.

### Fibre-optic probes with a small tip diameter can successfully be inserted into mouse lungs via the trachea

**Figure 3:**
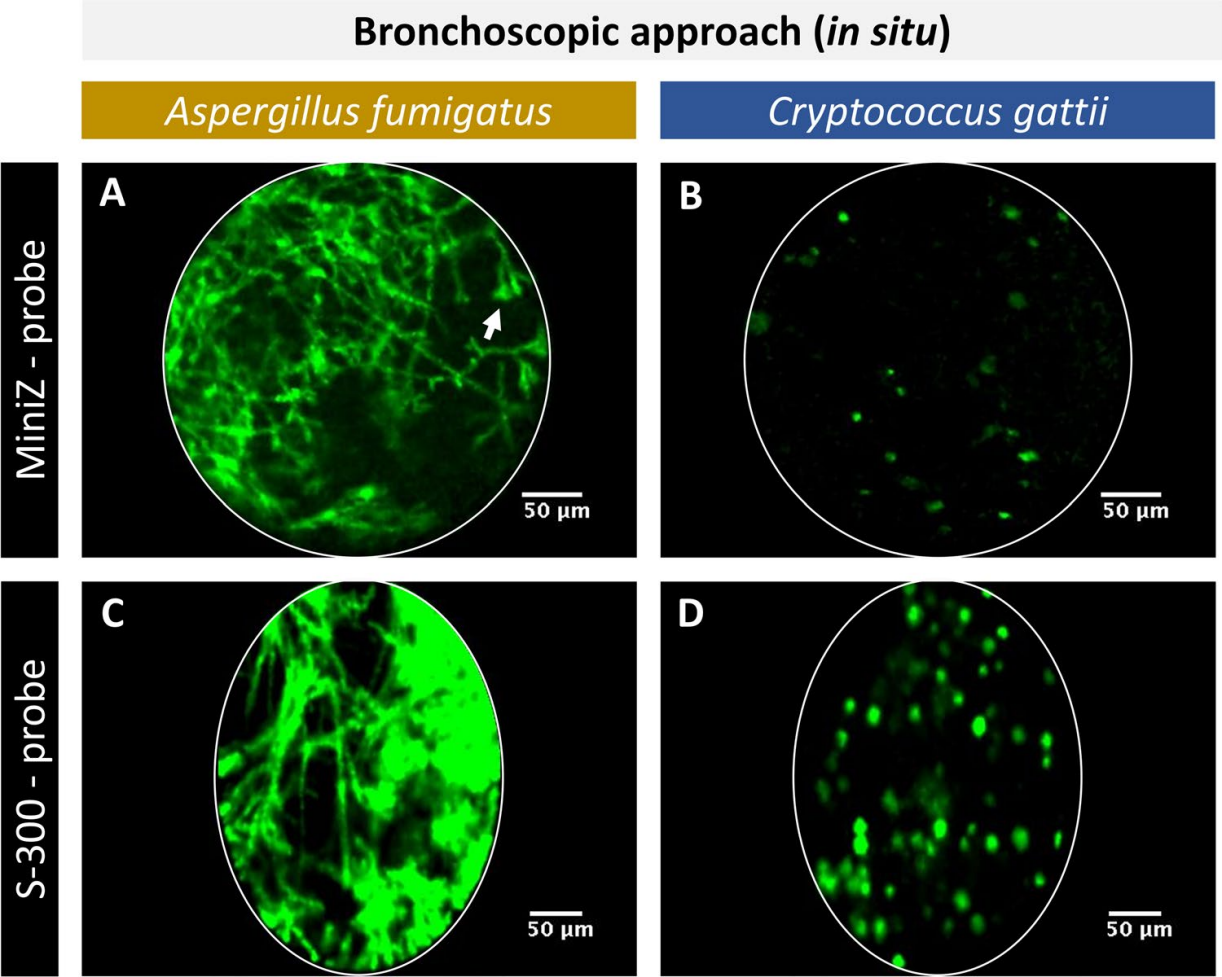
*In situ*, bronchoscopic FCFM of infected lungs using the Mini-Z or S-300 probe. The images were acquired by inserting the fibre-optic probe into the lung via a small incision in the trachea. (**A**,**B**) Representative FCFM images acquired with the Mini-Z probe in an *A*. *fumigatus* (GFP-expressing) infected lung and *C*. *gattii* (GFP-expressing) infected lung. (**C**,**D**) Representative FCFM images acquired with the S-300 probe in an *A*. *fumigatus* infected lung and a *C*. *gattii* infected lung showing clear differences in morphology for both species: densely packed hyphae and sporulating structures (white arrow) in case of *Aspergillus* infection, round cells in case of *Cryptococcus* infection.

Due to the invasiveness of transthoracic FCFM, it is not feasible to repeatedly apply this approach to follow up lung infection within the same animal. However, the use of fibre-optic probes with a small tip diameter could render this technique less invasive thanks to the possibility to endoscopically introduce the probe into the lungs. Several fibre-optic probes with different characteristics are commercially available, making each of them suitable for specific applications. In this study, the Mini-Z (0.94 mm tip diameter) was selected based on its advantageous characteristics such as an improved working distance (50 µm), which obviates the need for direct contact between the probe tip and target tissue. The feasibility of inserting this probe in the lungs was tested *in situ* after performing a bilateral thoracotomy on euthanized animals. The Mini-Z probe could successfully be inserted into the proximal parts of the mouse lung, including the trachea and bronchi, after tracheal exposure and incision. Images acquired in the lungs of *Aspergillus* infected animals showed the presence of densely packed hyphae and sporulating structures (white arrow) (Fig. 3A). Within the lungs of *C*. *gattii* infected animals, fewer cryptococcal cells were detected after bronchoscopic insertion of the Mini-Z probe compared to the transthoracic approach (Fig. 3B). However, due to the relatively large tip diameter (0.94 mm), it proved to be very difficult to manoeuvre the probe in the lungs and this would likely hamper respiration of the mouse during image acquisition. Therefore, this probe was not considered for further *in vivo* applications.

Subsequently, we tested whether the S-300 probe would be more suitable. Its smaller tip diameter (0.30 mm) allowed for a smooth entrance into the trachea and facilitated manoeuvring inside the lungs. Upon insertion, densely packed hyphae could be visualized within the lungs of *Aspergillus* infected animals (Fig. 3C) and a high number of cryptococcal cells were detected within the lungs of *C*. *gattii* infected animals (Fig. 3D).

### Bronchoscopic FCFM allows for longitudinal *in vivo* monitoring of fungal lung infections

Next, we investigated whether the abovementioned bronchoscopic approach was also feasible in an *in vivo* setting. The S-300 probe was selected based on its small diameter (0.30 mm), which facilitates its insertion into the lungs of the mouse via the mouth and trachea. To investigate potential damage inflicted to the lung tissue upon insertion of the fibre-optic probe, the animals were carefully monitored during and after the bronchoscopic FCFM procedure. The animals showed a quick recovery without visible signs of respiratory distress, proving that this approach can be used for *in vivo* FCFM imaging of the lungs without causing significant damage or distress to the animals.

**Figure 4:**
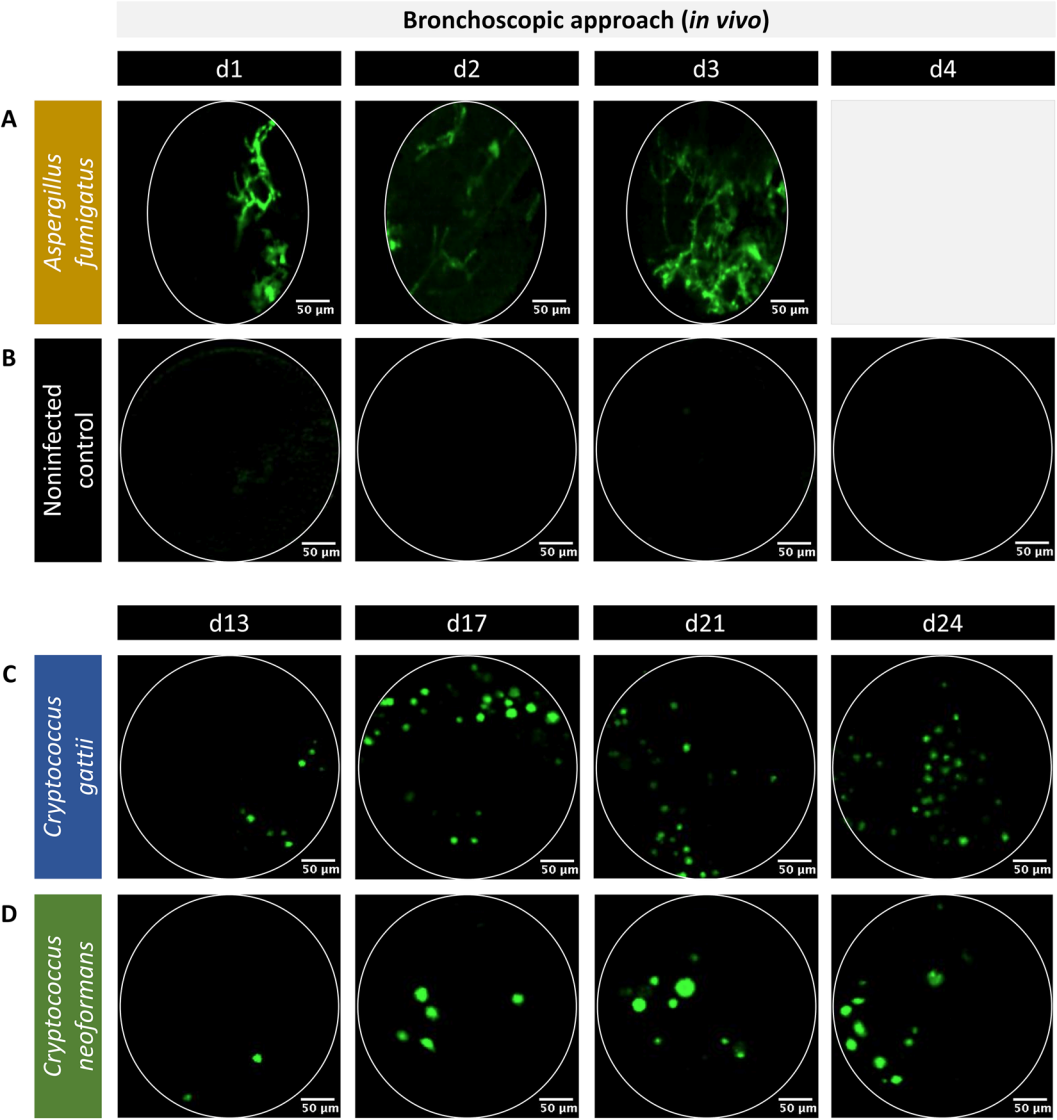
*In vivo* bronchoscopic FCFM for the longitudinal monitoring of fungal lung infections. The *in vivo* images were acquired by inserting the S-300 probe into the lungs of free-breathing, anesthetized animals via the mouth and trachea. All animals were intranasally infected with either a GFP-expressing *Aspergillus* or a GFP-expressing *Cryptococcus* strain. (**A**) Representative FCFM images of *A*. *fumigatus* infected lungs showed few hyphal structures on day 1 and 2 post infection, and a higher amount of hyphae on day 3. (**B**) Representative FCFM images of non-infected control animals were acquired on four consecutive days and showed no background signals. (**C**,**D**) In animals infected with *C*. *gattii* or *C*. *neoformans*, representative FCFM images acquired on day 13, 17, 21 and 24 post infection showed the presence of multiple round cells. In addition, a slightly larger cell size was observed for *C*. *neoformans* cells compared to the *C*. *gattii* cells.

To assess whether this approach is suitable for longitudinal monitoring of pulmonary fungal infections, an *in vivo* experiment was performed. Animals were imaged for three consecutive days in case of acute infection (aspergillosis) or at four time points during the course of slowly progressing infections (cryptococcosis), while non-infected mice served a negative controls. All animals tolerated the repeated bronchoscopic FCFM sessions well and the majority (94%) survived until the end of the experiment. Only one *Aspergillus* infected animal and one control animal died during the last imaging session on day 3 and day 4, respectively as a result of puncturing the lung. Within the lungs infected with GFP-expressing *Aspergillus*, few hyphal structures could be visualized upon probing the lung on day 1 and 2 post instillation (Fig. 4A). On day 3, relatively high amounts of hyphae were detectable compared to previous time points. In contrast, no signals could be observed in the lungs of non-infected control animals and animals infected with WT *Aspergillus* or WT *Cryptococcus* strains (Fig. 4B). Within the lungs of *C*. *gattii* infected animals, round cryptococcal cells were detectable starting from day 13 post instillation (Fig. 4C). Over time, a slight increase in the number of cryptococci was observed. Within the lungs of *C*. *neoformans* infected animals, few cryptococcal cells were detected on day 13 post instillation (Fig. 4D). The number of observed cells increased over time. Furthermore, the detected *C*. *neoformans* cells appeared to be larger in size compared to the *C*. *gattii* cells. Representative full image sequences were provided in the Supplementary Information for an *A*. *fumigatus* infected lung 3 days post instillation (Supplementary Video S5), a *C*. *gattii* infected lung 24 days post instillation (Supplementary Video S6) and a non-infected control lung (Supplementary Video S7).

### FCFM can provide quantitative information on pulmonary infections

**Figure 5:**
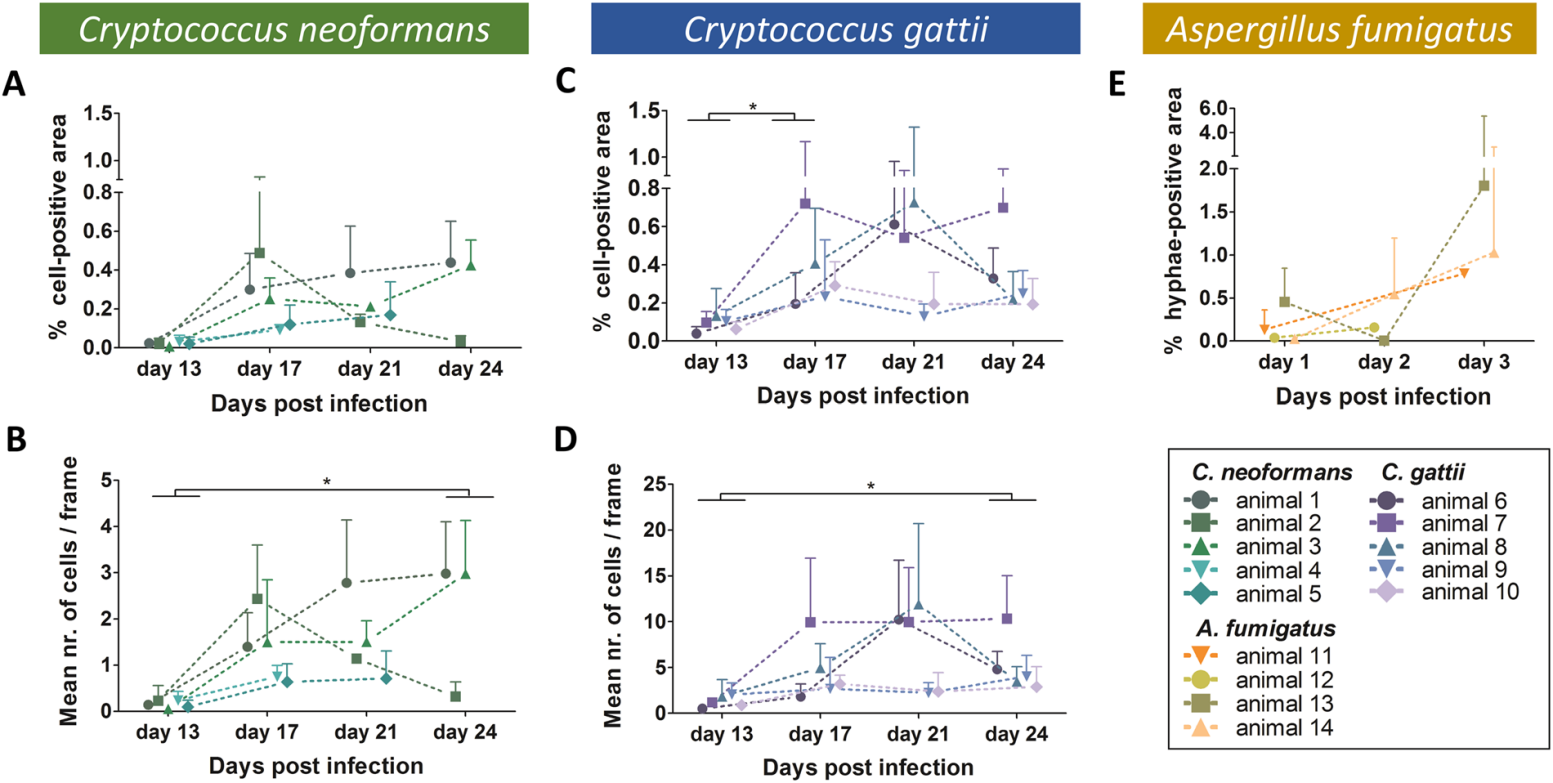
Quantitative analysis of the *in vivo* bronchoscopic FCFM images showing differences during the course of infection. For each animal, three different image sequences were acquired per time point. Subsequently, both the number of cells and the percentage cell-positive area per frame were quantified and averaged for each image sequence, resulting in three values per mouse per time point. (**A**–**D**) Graphs representing the percentage cell-positive area and mean number of cells per frame quantified for the individual *C*. *neoformans* infected animals (green, n = 5) or *C*. *gattii* infected animals (blue, n = 5), respectively. (**E**) Graph representing the percentage hyphae-positive area calculated for the *A*. *fumigatus* infected animals (n = 5). Data are shown as mean ± SD. Differences over time were analysed using a Friedman test (repeated measures) or a Kruskal-Wallis test (in case of missing values due to animal death or failed acquisition) with Dunn’s multiple comparison post-test (*p-value < 0.05).

To confirm our visual observations, all frames of the acquired bronchoscopic FCFM movies were semi-automatically analysed to obtain following readouts: percentage cell-positive area, number of cells and average cell size. To illustrate the quantitative analysis, representative graphs showing the percentage cell-positive area per frame and the mean of the imaging sequence are shown in Supplementary Figure 3. For the *Aspergillus* infected animals, the percentage hyphae-positive area clearly increased over time (Fig. 5E). Due to its characteristic hyphal growth, information about cell size or cell counts is not informative for this fungus. For the *C*. *neoformans* infected animals, the percentage cell-positive area and mean number of cells per frame was increased for all animals, except for mouse 2 (Fig. 5A,B). This animal showed a steep increase in both parameters on day 17, followed by a decrease on day 21 and day 24. All *C*. *gattii* infected animals displayed a trend towards increased percentages of cell-positive area and mean number of cells with time after infection (Fig. 5C,D). However, for some animals the values decreased again towards the last time point (animal 6 and 8). When comparing the results of *C*. *neoformans* and *C*. *gattii* infected animals, a larger number of cells per frame was detected for *C*. *gattii* infections, supporting our visual observations (Fig. 5B,D).

**Figure 6:**
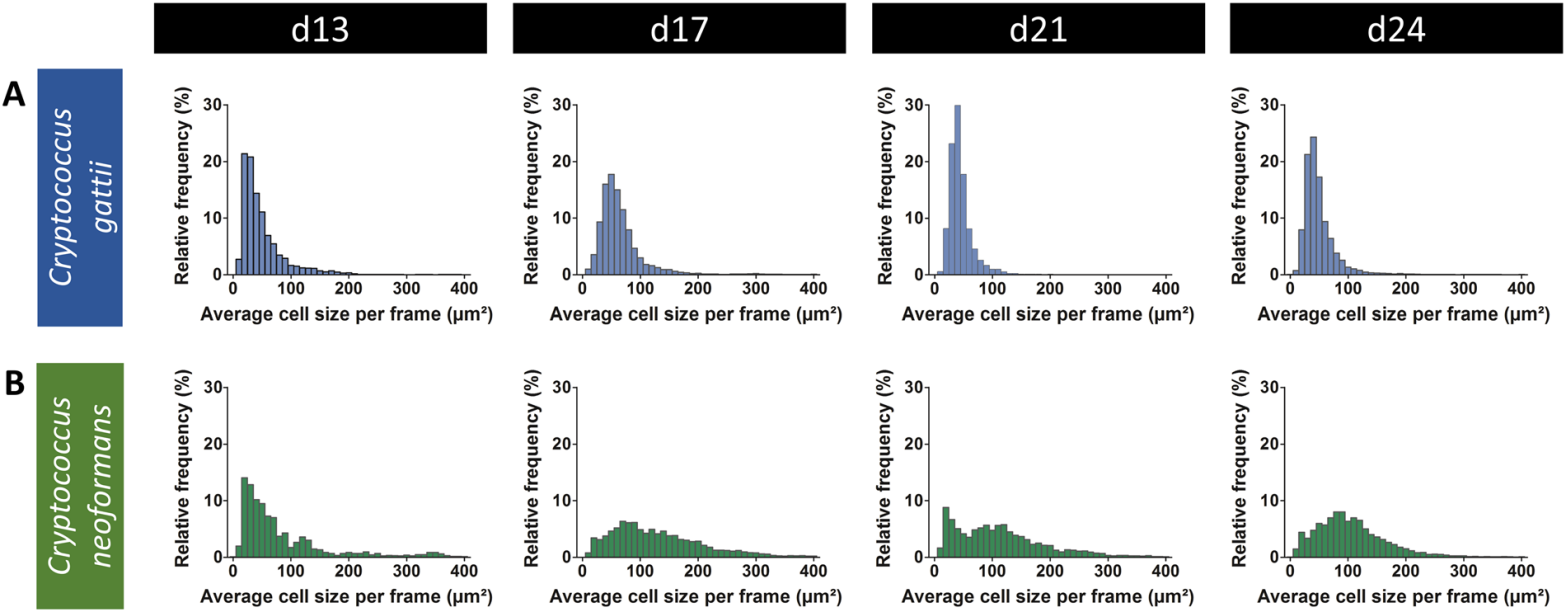
Quantitative analysis of the size distribution of cells detected on the *in vivo* bronchoscopic FCFM images. The average cell size per frame of all frames and animals were pooled per time point to obtain a relative frequency distribution of cell sizes. Frames containing zero detected cells or having a mean signal intensity lower than 5 were excluded from the analysis. Histograms (bin width of 10 µm²) representing the average cell size per frame on day 13, 17, 21 and 24 post instillation with *C*. *gattii* (top row) or *C*. *neoformans* (bottom row) show a wider variation in cell sizes for *C*. *neoformans*.

To obtain more profound insights into the differences in cell morphology between *C*. *gattii* and *C*. *neoformans in vivo*, the relative frequency distribution of the average cell size per frames was calculated. The resulting histograms showed a more extensive variability in cell sizes for the *C*. *neoformans* infected animals compared to the *C*. *gattii* infected animals (Fig. 6A,B). A larger cell size occurred more frequently in the *C*. *neoformans* infections. With regard to possible time-dependent evolutions in cells size, cell size distribution remained relatively constant for *C*. *gattii*. In contrast, the distribution for *C*. *neoforman*s was slightly shifted towards larger cell sizes.

### Bronchoscopic FCFM has a high sensitivity for the differential detection of fungal lung infections

To assess the suitability of the bronchoscopic FCFM imaging technique for the differential detection of fungal lung infections and for distinguishing them from control animals, the sensitivity and specificity were calculated. Four blinded observers classified all randomized image sequences into three categories: *Aspergillus*, *Cryptococcus* or control. The overall sensitivity to detect the induced fungal infection was calculated. The percentage of *C*. *gattii* infected animals that was correctly identified as *Cryptococcus* was higher for all observers (range 85–97%) compared to the sensitivity for the *C*. *neoformans* group (range 37–87%) (Table 1, bold columns). The sensitivity for the *Aspergillus* group was lower (range 34–51%) compared to both the *C*. *gattii* and *C*. *neoformans* group.

**Table 1:** Calculated sensitivity of bronchoscopic FCFM for detecting an induced fungal infection.

		Aspergillus fumigatus				Cryptococcus gattii				Cryptococcus neoformans					
		all	d1	d2	d3	all	d13	d17	d21	d24	all	d13	d17	d21	d24
sensitivity (%)	**observer 1**	**34**	19	45	50	**85**	60	87	94	87	**37**	0	50	94	67
	**observer 2**	**40**	31	45	50	**80**	60	87	81	100	**42**	0	63	50	67
	**observer 3**	**49**	38	64	50	**97**	87	100	100	100	**85**	60	88	100	100
	**observer 4**	**51**	50	55	50	**97**	87	100	100	100	**87**	60	94	100	100

Potential time-dependent differences were investigated by calculating the sensitivity for each time point. For the *Aspergillus* animals, the sensitivity increased over time for all observers, reaching a sensitivity of 50% for all observers by day 3. For both *Cryptococcus* groups the trend was similar, reaching a sensitivity of 100% by day 17 in the *C*. *gattii* group and by day 21 in the *C*. *neoformans* group for half of the observers. This time-dependent increase in correct classifications is consistent with the more frequent observation of fungal elements during the later stages of infection, as the probability of detecting an infected region will increase with progression of infection.

Finally, the specificity was calculated based on the classification results of the non-infected and WT-infected controls, including all image sequences from all time points. The proportion of correctly identified true negatives was high for all observers (range 80–98%) (Supplementary Table S4, bold column in bold) and consistent among the different time points.

On average, the sensitivities obtained from the results of observer 4 were higher compared to the other observers, showing that prior experience with the infection models and involvement in image acquisition result in an increased success rate during classification. In contrast, the specificity was slightly higher for the untrained observers, except for observer 3. Nevertheless, an overall substantial inter-observer agreement was obtained between all observers with Fleiss’ kappa of 0.67.

### Histological analysis of the lungs confirms infection and shows no significant induction of damage following bronchoscopic FCFM

**Figure 7:**
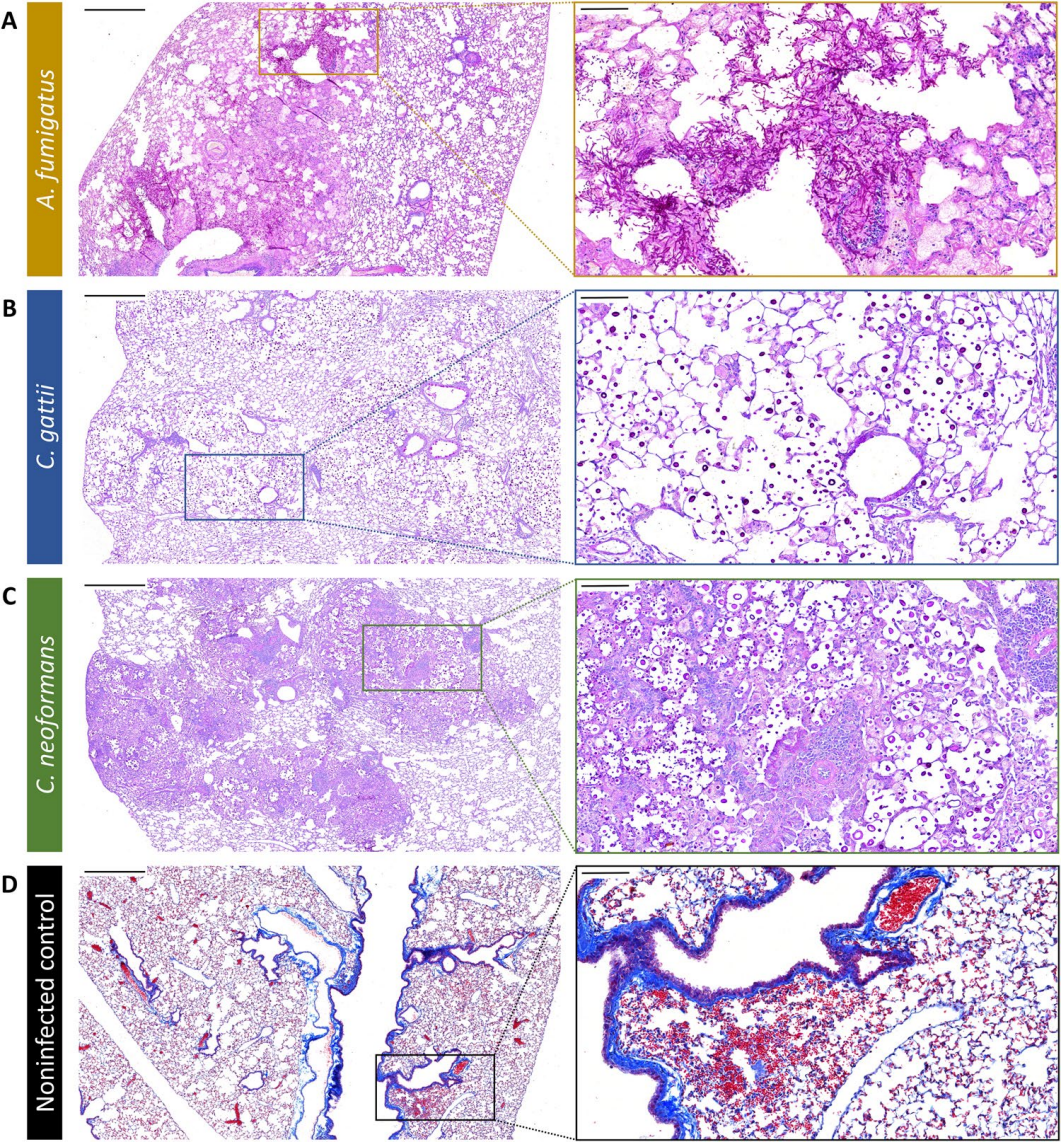
Validation of the *in vivo* bronchoscopic FCFM results by histology. Following the last imaging time point, all animals were sacrificed and the lungs were isolated for histological analysis. (**A**–**C**) Representative light microscopy images (Periodic Acid-Schiff staining) showing an *A*. *fumigatus*, *C*. *gattii* or *C*. *neoformans* infected lung section, confirming infection in the imaged animals. (**D**) Representative light microscopy images from a non-infected control animal showing small haemorrhages within the lung tissue (Masson’s Trichrome staining). Scale bars measure 500 µm (left) or 100 µm (right).

After the last imaging time point, histology was performed to validate the bronchoscopic FCFM results. In *A*. *fumigatus* infected lungs, large amounts of hyphal structures were present, which mainly accumulated around the major airways (Fig. 7A). These hyphae invaded both the bronchi and alveolar spaces, thereby disrupting the normal pulmonary structure. Lungs infected with *C*. *gattii* showed nodular lesions throughout the lungs (Fig. 7B). Detailed examination revealed the presence of high numbers of cryptococcal cells in the alveolar spaces, without apparent disruption of the lung structure. Similarly, multiple nodular lesions were observed in *C*. *neoformans* infected lungs (Fig. 7C). In contrast with *C*. *gattii*, these lesions were characterized by a prominent infiltration of inflammatory cells and damage to the normal lung structure. Furthermore, there seemed to be an increased variability in cell size compared to *C*. *gattii*, which is consistent with our visual and quantitative observations following bronchoscopic FCFM in both *Cryptococcus* models.

To investigate potential probe-induced lung damage caused by the bronchoscopic procedure, non-infected control animals were imaged with either a blunt or bevelled tip (45°) fibre-optic probe. After the last imaging session, the lungs were examined on both a macroscopic and microscopic level. Upon visual inspection of the lungs, no apparent signs of damage could be detected. However, histological examination revealed the presence of multiple small haemorrhages within the lungs of all control animals (Fig. 7D). The percentages of affected lung area calculated from animals imaged with the blunt probe (0.21% ± 0.31) or bevelled probe (0.35% ± 0.43) were not significantly different. Haemorrhage affected only a small percentage of the total lung area and no respiratory distress was detected, indicating that bronchoscopic FCFM is minimally invasive.

## Discussion

FCFM is a relatively new optical imaging technique that allows for the real-time detection of fluorescently labelled cells within tissues of living animals^19^. The lungs can be imaged either by exposing the lung surface (transthoracic) or by entering the lungs via a natural opening (endoscopic). The latter is preferred as it circumvents the need for a surgical procedure and thus strongly decreases the invasiveness of the procedure. However, the dimensions of the available fibre-optic probes do not always allow for endoscopic insertion, especially in small laboratory animals. In our study, we explored the suitability of various commercially available fibre-optic probes with different specifications for FCFM lung imaging in the mouse, as this animal is frequently used as a model for various respiratory diseases. To the best of our knowledge, we are the first to deliver an optimized FCFM imaging procedure that allows for repeated microscopic detection of fungal infections in mouse lungs *in vivo* in a minimally invasive manner.

Fibre-optic probes with a tip diameter exceeding 1 mm (such as the S-1500 probe) are unfit for endoscopic insertion into the mouse lungs and require a transthoracic approach to enable lung imaging. Due to the limited working distance of the S-1500 probe, the focal point is situated at 0 µm from the tip of the probe. Therefore, the fluorescently labelled cells need to be located within a close range of the lung surface in order to be visualized. The superficial location of cryptococcal lesions allowed for a close contact between the lesion and the probe, thereby increasing the success rate for visualizing cryptococci by using transthoracic FCFM. However, deeply located *Aspergillus* infections could not properly be visualized via the transthoracic approach, emphasizing the need for a bronchoscopic approach to enable imaging of inner lung structures. Subsequently, we investigated whether fibre-optic probes with a tip diameter smaller than 1 mm are suitable for bronchoscopic insertion into mouse lungs.

Only the S-300 probe with a diameter of 0.3 mm was found small enough to enable smooth insertion via the trachea and allowed for imaging of both the proximal and distal areas of the mouse lung, thereby leading to the successful visualization of individual fungal cells in both infection models. Both on a macroscopic and microscopic scale, no significant damage to the lung tissue was found, regardless of the probes’ tip shape. These results show that the S-300 probe can safely be used to image mouse lungs in a bronchoscopic approach. Previously, only a limited number of studies have applied a similar technique to image lung inflammation and regeneration in mice^17,18^. One of these studies reported on repeated lung FCFM imaging on the same animal^18^. However, in these studies the animals were intubated and ventilated during image acquisition. Although this can allow for additional lung function measurements, it requires specialized equipment and expertise. In this study, the small probe diameter enabled us to demonstrate the feasibility of performing repeated FCFM measurements in the lungs of free-breathing mice, thereby allowing for a physiologically relevant longitudinal assessment of pulmonary diseases.

The power of this technique for detecting fungal lung infections was evaluated based on the results of a classification task. At each time point, the sensitivity of detection was highest for the *C*. *gattii* infected group. This can be explained by the frequent observation of multiple round cells within one frame at both the early and late stages of infection. In contrast, the lower sensitivities obtained at the early time points for the *C*. *neoformans* group are consistent with the very low number of cells observed at this stage. For the *Aspergillus* infected animals, the obtained sensitivity was generally lower due to large inter-animal variability, as previously reported for this animal model^6^. In addition, it proved to be more challenging to bring the *Aspergillus* hyphae into focus. The calculated specificity was high for most observers, indicating that healthy or animals infected with WT fungi can clearly be distinguished from animals infected with GFP-expressing fungal strains. Moreover, it proved to be possible to correctly classify *Aspergillus* or *Cryptococcus* infected animals based on the differences in morphological appearance.

Besides the visual assessment of disease progression in the different fungal infection models, we also succeeded in obtaining quantitative readouts on the evolution of infection for each individual animal. For most animals, both the percentage cell-positive area and number of cells increased with time, which is likely associated with the progression of infection. However, considerable variability was observed, as the signal is highly dependent on the extent of infection in the imaged lung region and on whether or not a successful contact between the fungal cells and the probe was made. With the current FCFM technique, it is not possible to obtain detailed information on the exact positioning of the probe in the anatomical context of the infected lung. In our study, we aimed to minimize the effect of regional variability by acquiring and analysing three different image sequences per animal per time point, thereby averaging potentially extreme values. Hereby, one must be aware that this method might result in repeated imaging and thus repeated quantification of certain cells. A potential solution to obtain information about the positioning of the probe would be to combine FCFM with white light bronchoscopy, but this remains challenging despite advances in developing smaller bronchoscopes for mice^20^. An alternative solution would be to combine bronchoscopic techniques with other, anatomical imaging techniques such as magnetic resonance imaging or computed tomography (CT), as previously demonstrated in larger animal models such as rabbits^21,22^. However, initial experiments in our mouse models indicated that such a combinatory approach is technically challenging.

In this study, we succeeded in documenting morphological differences between two cryptococcal strains from two different species: *C*. *gattii* cells were typically smaller, whereas *C*. *neoformans* cells show a wider variation in cell sizes. Relatively large cryptococcal cells could occasionally be observed in the *C*. *neoformans* infected lungs, indicating a possible *in vivo* observation of the previously described titan cells^23,24^. In future studies, bronchoscopic FCFM might provide additional insights in the pathogenesis of infection. Moreover, this technique could allow more detailed *in vivo* studies of host-pathogen interactions by using an FCFM system with a second channel operating at a different wavelength. This could allow the visualization of host cells or structures tagged with a different fluorophore. In addition to the infectious diseases field, bronchoscopic FCFM could be of added value to assess biological questions in a variety of lung disease models.

In current clinical practice, bronchoscopic examination is a standard technique to investigate lung diseases. Recently, there has been an increasing interest in incorporating a fibre-optic probe into these bronchoscopes, as this allows real-time microscopic examination of lung tissue. Previous clinical studies have shown that this approach can successfully be used to investigate the human airways and lungs based on autofluorescence originating from the elastin network^25–28^. To date, applications in the field of fungal lung infections have been limited because of a lack of specific signals originating from the pathogens^29,30^. One possible solution to solve the lack of specificity in WT infections is the *in vivo* use of fluorophores that specifically bind to fungal elements. The development of these fluorophores is an active area of research, but is at the moment mainly limited to *in vitro* studies^31–33^. Our bronchoscopic FCFM approach could contribute to the preclinical, *in vivo* evaluation of these fluorophores. Future availability of targeted fluorophores for clinical use could contribute to improve the differential diagnosis of fungal infections.

In conclusion, we demonstrated the feasibility of performing repeated bronchoscopic *in vivo* FCFM for the assessment of pulmonary infections in murine models. Our minimally invasive bronchoscopic approach allowed for longitudinal studies in individual animals, thereby gaining quantitative information about the progression of infection with cellular resolution. In future studies, this technique might provide additional insights in the pathogenesis of infection and host-pathogen interactions. As bronchoscopic FCFM can readily be applied to study a variety of other pulmonary diseases, this work provides a novel approach that allows for detailed microscopic studies of the lung in free-breathing animals. Hereby, this technique can bridge the gap between histological studies and other preclinical *in vivo* imaging methods.

## Materials and Methods

### Fungal strains

A green fluorescent protein (GFP)-expressing *Aspergillus fumigatus* strain (FGSC A1258) was obtained from the fungal genetic stock centre (Kansas City, Missouri, USA). A wild type (WT) *A*. *fumigatus* strain (ASFU 1731) was originally isolated from a patient suffering from invasive aspergillosis (University Hospitals (UZ) Leuven, Belgium). The fungi were cultured on diluted Sabouraud agar for three days at 42 °C. Conidia were harvested by flooding the agar with 0.1% Tween − 80 (Sigma-Aldrich, Diegem, Belgium) in saline and gently scraping the surface. The collected suspension was shaken for 5 minutes. Spores were counted with a Neubauer haemocytometer and diluted in saline to a final concentration of 2.5 × 10^7^ spores/ml.

GFP-expressing or WT *Cryptococcus gattii* (R265) and *C*. *neoformans* (H99) strains^34^ were cultured on Sabouraud agar for 4 days at 30 °C. After gently scraping the cryptococcal cells from the agar, the cells were diluted in phosphate-buffered saline (PBS, Gibco, Parsley, UK) to a final concentration of 1.25 × 10^7^ cells/ml. For induction of the *in vivo* infection model, the inoculum size was confirmed by performing colony-forming unit (CFU) counts of the suspensions.

### Mouse models

All animal experiments were carried out in compliance with national and European regulations and were approved by the animal ethics committee of KU Leuven. Ten-week old male BALB/c mice were used for the *Aspergillus* infection model. To induce neutropenia, the animals received intraperitoneal (IP) injections of cyclophosphamide (150 mg/kg, Sigma-Aldrich, Diegem, Belgium) on day 4 and day 1 prior to infection. A broad-spectrum antibiotic (Baytril^®^, 5–8 mg/kg/day) was added to the drinking water to prevent bacterial infection. The animals were intranasally instilled with 20 µl of a suspension containing 5 × 10^5^ spores of either a WT (n = 3) or GFP-expressing (n = 5) *A*. *fumigatus* strain. For the *Cryptococcus* infection model, ten-week old female BALB/c mice were intranasally instilled with 20 µl of a suspension containing 2.5 × 10^5^ *C*. *gattii* or *C*. *neoformans* cells (GFP-expressing (n = 5 per strain) or WT (n = 3 per strain)). Following instillation, the animals were held in an upright position until normal breathing was resumed. Healthy, uninfected animals were used as negative controls (n = 5 per S-300 probe tested).

### Fibered Confocal Fluorescence Microscopy (FCFM)

FCFM imaging was performed by using a Cellvizio® system (Mauna Kea Technologies, Paris, France) with an excitation wavelength of 488 nm and a collection bandwidth of 505–700 nm. FCFM images were acquired with three different fibre-optic probes: the S-1500, Mini-Z and S-300 probe. Specifications and characteristics of the probes can be found in the Supplementary Methods. In healthy controls, the S-300 with either bevelled (45°) or blunt tip was used to study differences in potential lung damage caused by the *in vivo* imaging protocol.

#### *In vitro* FCFM

Fungal colonies were obtained by plating 100 µl of a fungal suspension containing *A*. *fumigatus* spores or *C*. *gattii* cells (GFP-expressing or WT strains) on Sabouraud agar, followed by 2 days of incubation at 30 °C. FCFM images were acquired by making direct contact between the fibre-optic probe (S-1500) and the surface of the fungal colonies or the uninfected surface of the agar.

#### *In situ* FCFM, transthoracic approach

The animals were deeply anesthetized by IP injection of 15 mg pentobarbital (Nembutal®, CEVA Santé Animale, Diegem, Belgium) and fixed in a supine position. Subsequently, the lungs were exposed by performing a bilateral thoracotomy. FCFM images were acquired by placing the probe (S-1500) directly against infected areas. As negative controls, macroscopically healthy appearing areas of infected lungs and lungs of non-infected mice (n = 3) were imaged. *Aspergillus* infected animals (n = 4) were imaged 4 days after infection. For the *Cryptococcus* model, animals (n = 2) were infected with 5 × 10^4^ *C*. *gattii* cells and imaged 15 days after infection. All animals were sacrificed after the image acquisitions.

#### *In situ* FCFM, endoscopic approach

Mice were euthanized using an overdose of pentobarbital. Next, the lungs were exposed by performing a bilateral thoracotomy to allow for visual assessment of the probe’s position during the imaging session. Images were acquired by entering the lungs with the fibre-optic probe (S-300 or Mini-Z) via a small incision in the trachea. The *Aspergillus* infected animals were imaged on day 4 after infection and the *Cryptococcus* infected animals were imaged on day 12 (Mini-Z probe) or day 14 (S-300 probe) post infection.

#### *In vivo* FCFM, endoscopic approach

Animals were anesthetized by an IP injection of a ketamine (45–60 mg/kg, Nimatek®, Eurovet Animal Health, Bladel, The Netherlands) - medetomidine (0.6–0.8 mg/kg, Domitor®, Elanco Animal Health, Brussels, Belgium) mixture, supplemented with atropine sulphate (0.375–0.485 mg/kg, Sterop, Brussels, Belgium) to counteract the swallowing reflex and salivation. Eye gel (Vidisic®, Bausch & Lomb Pharma, Brussels, Belgium) was applied to prevent the eyes from drying out. The animals were fixed in supine position on top of a heating pad to maintain a constant body temperature. To gain easy access to the throat, a small thread was applied behind the front teeth and attached to the heating plate. Subsequently, the tongue was gently pulled sideways and the lower jaw was lifted to obtain a clear view on the tracheal entrance. A LED illuminator (Fiber-Lite®, Dolan-Jenner Industries, Massachusetts, United States) was focused on the throat of the animal to increase the visibility at the tracheal entrance. Next, the inactivated fibre-optic probe (S-300 probe, bevelled or blunt) was gently inserted via the trachea into the lungs. Whenever resistance was felt during insertion, the probe was slightly retracted to prevent puncturing the tissue. FCFM images were acquired while carefully manoeuvring the probe within different parts of both the left and right lungs. The position of the S-300 probe within the lungs could roughly be estimated based on the location of the visible light emitted from the tip of the probe and on the depth of insertion. During acquisition, the animal’s breathing rate was monitored by visual inspection. For each animal, three separate image sequences of approximately 1.5 minutes were acquired at every time point. In between these acquisitions, the optical probe was completely retracted from the lungs, checked for potential residual signal and cleaned when necessary. After the last acquisition, anaesthesia was reversed by IP injection of atipamezole hydrochloride (0.5 mg/kg, Antisedan®, Orion pharma, Espoo, Finland) and the animals were allowed to recover on a heating plate. A flow chart of the bronchoscopic FCFM procedure, illustrating all steps from animal infection to image analysis can be found in Supplementary Figure 1. Furthermore, a demonstration of the bronchoscopic FCFM procedure is provided in the Supplementary Information (Supplementary Figure S2 and Supplementary Video S8).

*Aspergillus* infected animals (n = 5 for GFP-expressing strain, n = 3 for WT strain) were imaged on day 1, 2 and 3 after infection. *C*. *gattii* and *C*. *neoformans* infected animals (n = 5 for GFP-expressing strains, n = 3 for WT strains) were imaged on day 13, 17, 21 and 24 after infection. All *in vivo* images were acquired using the blunt S-300 probe (circular field of view), except for the GFP^+^ *Aspergillus* infected animals, which were imaged using the bevelled S-300 probe (oval field of view). Healthy control animals (n = 4 bevelled S-300 probe, n = 5 blunt S-300 probe) were imaged on four consecutive days. For each animal, three separate image sequences of approximately 1.5 minutes were acquired at every time point.

#### Image analysis

Images were quantified using ImageJ (version 1.49, National Institutes of Health, USA). Following parameters were obtained: signal intensity, percentage cell-positive area, number of cells and average cell size per frame. To illustrate the quantitative analysis, representative graphs showing the percentage cell-positive area per frame of a full imaging sequence are shown in Supplementary Figure 3. The number of cells and percentage cell-positive area per frame were averaged per image sequence, leading to three values per mouse per time point. Details of the quantification algorithm can be found in the Supplementary Methods.

To assess the sensitivity, or true positive rate, for detecting an induced fungal infection and its specificity to identify negative controls (either WT-infected or noninfected), sequences acquired with the *in vivo*, endoscopic protocol were randomized and classified by four blinded observers, as described in the Supplementary Methods. The sensitivity was calculated as the proportion of correctly identified infected animals for each infection group seperately. The specificity was calculated as the proportion of correctly classified control animals (WT infected or noninfected). Additionally, sensitivity and specificity were calculated for the different time points to assess potential differences over time.

### Histological analysis

Following the last *in vivo* image acquisition, animals were euthanized by an overdose of pentobarbital. Subsequently, the lungs were isolated and formalin-fixed. Paraffin-embedded tissue sections were stained using a Masson’s trichrome (MTC) or periodic acid-Schiff (PAS) staining. Details on the histological procedures can be found in the Supplementary Methods.

### Statistics

Statistical analysis was performed using GraphPad PRISM (version 5.04, San Diego, CA, USA). Differences over time were analysed using a Friedman test (repeated measures) with Dunn’s post hoc test (two-tailed with α of 0.05). In case of missing values (*Aspergillus* and *C*. *neoformans* data), a Kruskal-Wallis test was used. Data were presented as mean ± standard deviation (SD). To calculate the average cell sizes per frame, data from all animals were pooled per time point, with exclusion of the frames where zero cells were detected. Relative frequency distributions were determined using GraphPad PRISM to make histograms with a bin width of 10 µm².

### Data availability

All data generated or analysed during this study are available from the corresponding author on reasonable request.

## Electronic supplementary material

Supplementary Information

Supplementary Video S5

Supplementary Video S6

Supplementary Video S7

Supplementary Video S8
