# Supplementary material for: Bronchoscopic fibered confocal fluorescence microscopy for longitudinal in vivo assessment of pulmonary fungal infections in free-breathing mice
Source: Sci Rep. 2018 Feb 14;8:3009. doi: 10.1038/s41598-018-20545-4 (PMC5813038; doi:10.1038/s41598-018-20545-4)
Supplement: Supplementary file 1 — Supplementary Information [file 41598_2018_20545_MOESM1_ESM.doc]

**SUPPLEMENTARY INFORMATION**

**Full title**

Bronchoscopic fibered confocal fluorescence microscopy for longitudinal in vivo assessment of pulmonary fungal infections in free-breathing mice

**Authors**

Liesbeth Vanherp^1#^, Jennifer Poelmans^1#^, Amy Hillen^1^, Kristof Govaerts^1^, Sarah Belderbos^1^, Tinne Buelens^1^, Katrien Lagrou^2^, Uwe Himmelreich^1^, Greetje Vande Velde^1*^

**Affiliations**

^1^ Biomedical MRI unit/ MoSAIC, Department of Imaging and Pathology, KU Leuven, Herestraat 49 O&N1 box 505, 3000 Leuven, Belgium;

^2^ Laboratory of Clinical Bacteriology and Mycology, Department of Microbiology and Immunology, KU Leuven, Herestraat 49 box 6711, 3000 Leuven, Belgium.

^#^ L.V. and J.P. contributed equally as first authors.

^*^ Correspondence: [greetje.vandevelde@kuleuven.be](mailto:greetje.vandevelde@kuleuven.be)

**SUPPLEMENTARY METHODS**

**Fibre-optic probes**

The S-1500 probe was used for the in vitro and transthoracic measurements and has following characteristics: 1.5 mm tip diameter, 3.3 µm lateral resolution, 15 µm optical sectioning, 0 µm working distance and 600 µm maximum field of view (FOV). The Mini-Z probe was used for ex vivo endoscopic measurements and has following characteristics: 0.94 mm tip diameter, 3.5 µm lateral resolution, 30 µm optical sectioning, 50 µm working distance and 325 µm maximum FOV. The S-300 probe was used for in vivo and ex vivo endoscopic measurements and has following characteristics: 0.3 mm tip diameter, 3.3 µm lateral resolution, 15 µm optical sectioning, 0 µm working distance and 300 µm maximum FOV.

**FCFM Image Analysis**

After acquisition, all frames of the image sequences were exported as JPEG files using ImageCell software (version 3.2.0, Mauna Kea Technologies, Paris, France). Subsequently, they were viewed and analysed as hyperstacks in ImageJ (version 1.49, National Institutes of Health, USA). A quantitative analysis of the in vivo results was performed using an in-house written macro in ImageJ, similar to a previously published and validated algorithm for quantifying FCFM data 35. For every image sequence, the field-of-view (FOV) of all frames was analysed by using an automated thresholding algorithm (Moments Dark algorithm 36 for Cryptococcus data, IsoData Dark algorithm 37 for Aspergillus data). Subsequently, the ‘analyse particles’ tool was used with the following settings: circularity (0.05 - 1.00 for Cryptococcus, 0.00 - 0.50 for Aspergillus) and size (0 - max size of FOV for both fungal species). Following parameters were obtained: signal intensity, percentage cell-positive area, number of cells and average cell size per frame. To account for the incorrect detection of signals that are not visible upon visual inspection, frames with mean signal intensities lower than five were manually corrected to zero counts and zero percentage cell-positive area. In addition, the quantification results were compared with a visual inspection of the images to correct for potential errors introduced by automatic quantification, thereby excluding frames when discordances were detected. The number of cells and percentage cell-positive area per frame were averaged per image sequence, leading to three values per mouse per time point

To calculate the sensitivity and specificity, four independent, blinded observers were asked to classify each movie into three categories (Aspergillus, Cryptococcus or control) based on the morphological appearance of the detected signal (round structures for Cryptococcus, filamentous structures for Aspergillus and the absence of signal for control). One of the observers (observer 4) was involved in the image acquisition and had prior experience with the infection models, while the other three observers (observer 1 - 3) were only informed about the morphological appearance of each fungal species based on an example image. A Fleiss’ kappa was calculated to assess agreement between the different observers.

**Histology**

For the non-infected control mice, lungs were inflated with 0.7 ml 10 % - formalin by inserting a 22-gauge catheter in the trachea. For both the *Aspergillus* and *Cryptococcus* infected animals, only the left lung was inflated with 0.5 ml formalin (10 % in PBS). Subsequently, the lungs were isolated and post-fixated in 10 % formalin for 24 hours at 4°C. Then, the tissue was embedded in paraffin and sectioned (5 µm). To check for damage, the lungs of the non-infected control animals were stained with Masson's trichrome (MTC), which stains collagen blue and erythrocytes red. To confirm successful infection, the left lung of the infected animals was stained with periodic acid-Schiff (PAS), which stains fungi purple.

The stained lung sections were scanned using a Mirax Desk (Zeiss, Göttingen, Germany) and Panoramic Viewer (version 1.15.4, 3DHISTECH, Budapest, Hungary) was used to view the slides digitally. For both fungal species, two sections per lung were visually examined to confirm infection in all animals. For the non-infected controls, 10 consecutive sections per lung were visually inspected to identify damaged regions. These regions were manually delineated and the surface area was multiplied with the intersection thickness to determine the volume of probe-induced injury. Afterwards, the total lung area was quantified for each section by delineating the complete lung in ImageJ. The total lung volume was calculated by multiplying the quantified area by the intersection thickness. For each lung, the ratio of injury volume to total lung volume was calculated.

**SUPPLEMENTARY FIGURES**

**
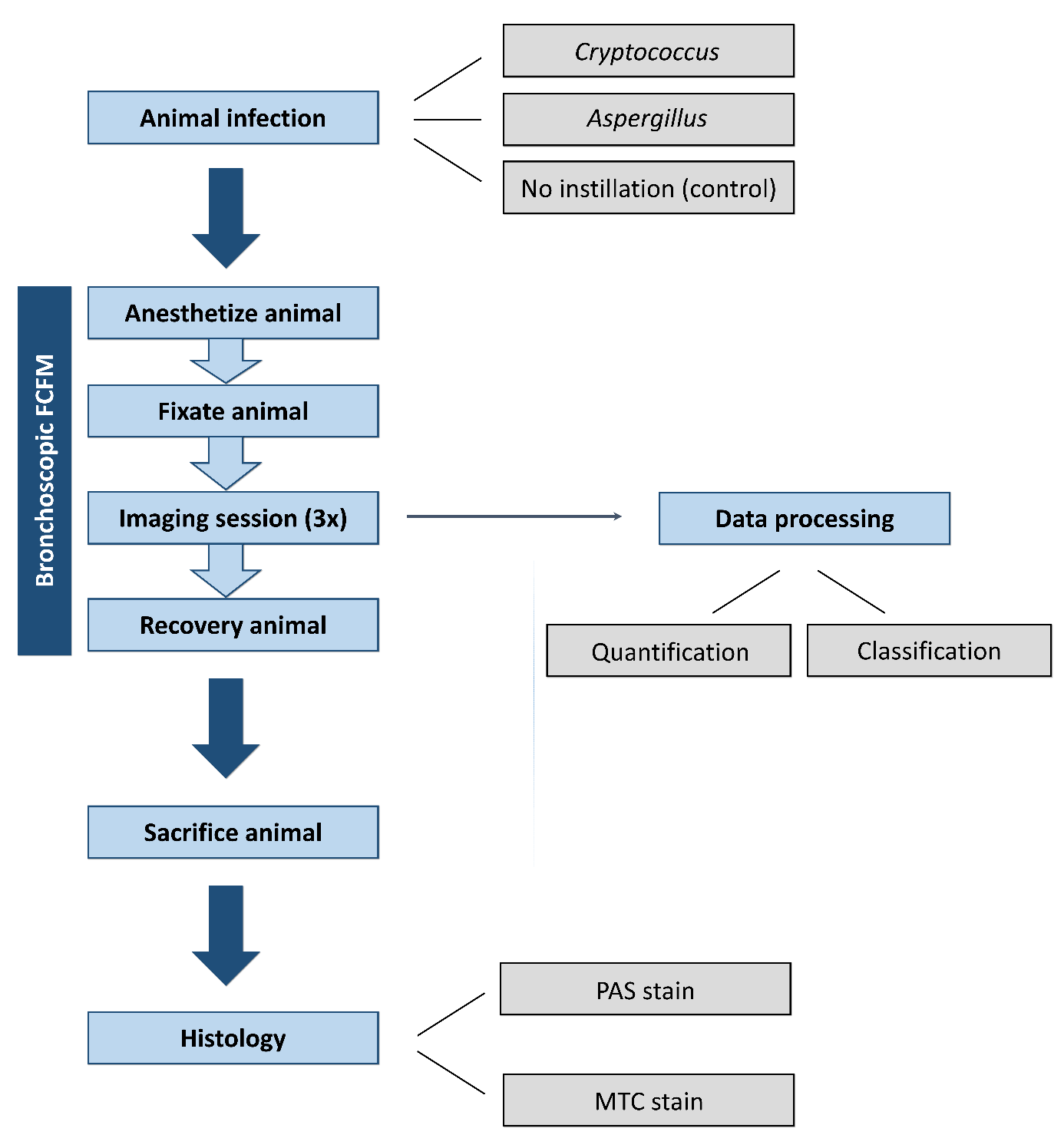
**

**Supplementary Figure S1:** Flow chart of the bronchoscopic FCFM workflow, including model induction, imaging procedure and data analysis.


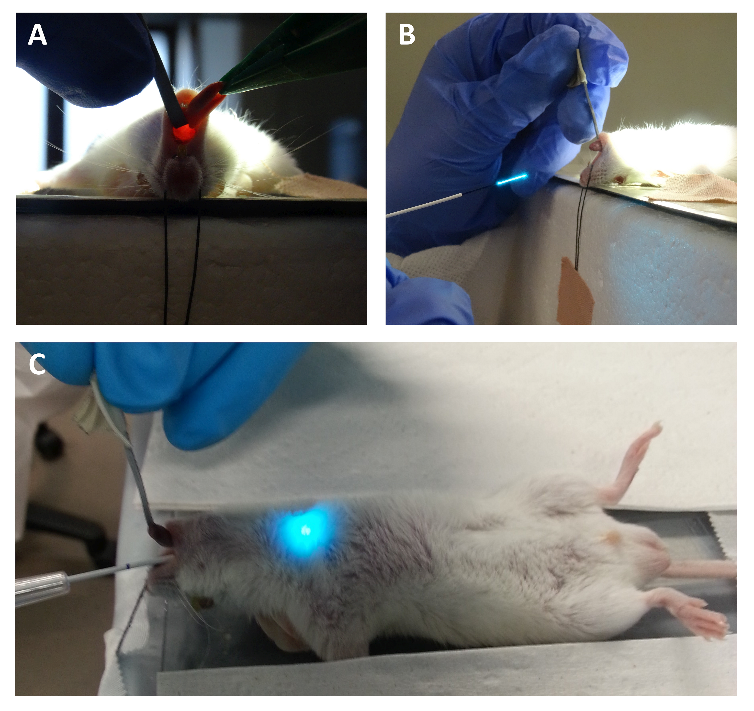


**Supplementary Figure S2: Experimental setup for *in vivo* bronchoscopic FCFM.** Before start of the procedure, the mice were deeply anesthetized and fixed in a supine position. (A) To gain easy access to the throat, a small thread was applied behind the frontal teeth. Subsequently, the lower jaw was lifted and the tongue was gently pulled aside. Visibility at the tracheal entrance was further increased by focusing an illuminator on the throat. (B) The S-300 probe was gently inserted into the lungs via the mouth and trachea without activating the laser (switched on in the picture for demonstration purposes). The laser was activated and the acquisitions started when the probe was located inside the lungs. (C) FCFM images were acquired by carefully manoeuvring the S-300 probe within different parts of the left and right lungs. The position of the probe could be roughly estimated based on the location of the visible light emitted by the tip.

**
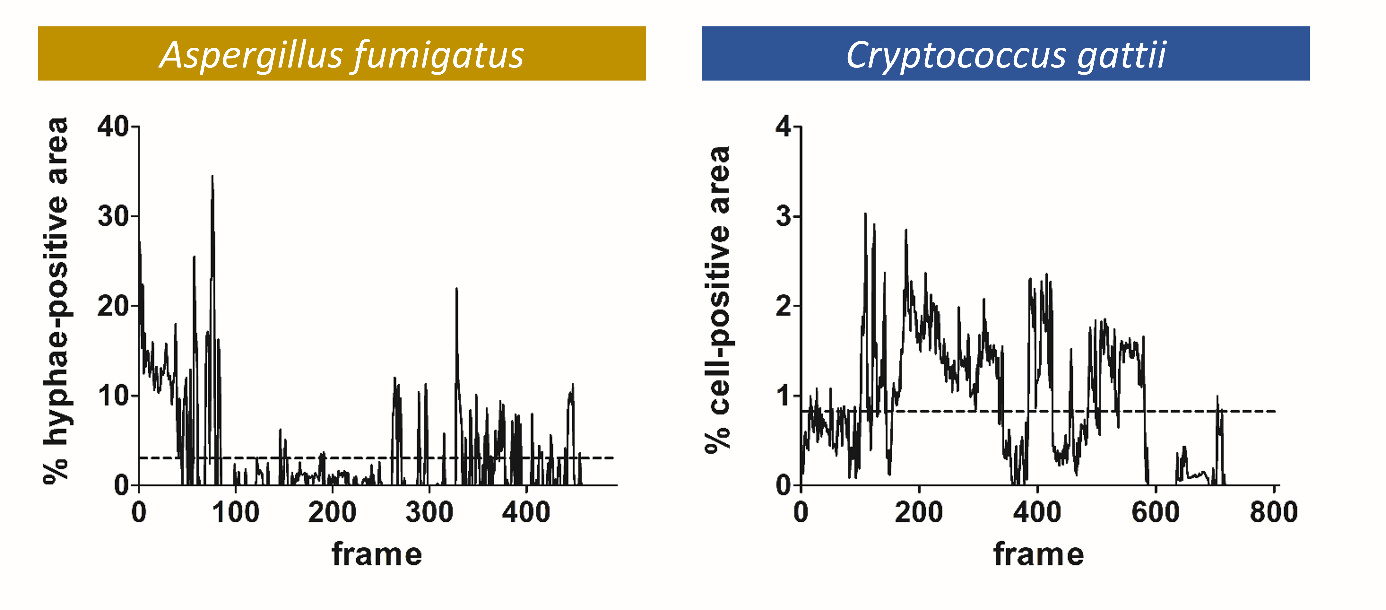
Supplementary Figure S3: Quantitative analysis of a full imaging sequence of an *A. fumigatus* and *C. gattii* infected lung.** These graphs show the percentage cell-positive area as quantified for each frame of the full imaging sequence of an *A. fumigatus* infected lung (Supplementary video S4) and *C. gattii* infected lung (Supplementary video S5). The dashed line shows the calculated mean of the imaging sequence that was subsequently used as a representative value for the full imaging sequence.

**SUPPLEMENTARY TABLE S4**

Calculated specificity of bronchoscopic FCFM for identifying negative controls.

|  | | **WT and non-infected controls** | | | | |
| --- | --- | --- | --- | --- | --- | --- |
|  |  | **all** | **d1** | **d2** | **d3** | **d4** |
| **specificity (%)** | **observer 1** | **98** | 100 | 100 | 97 | 92 |
|  | **observer 2** | **98** | 97 | 100 | 97 | 96 |
|  | **observer 3** | **80** | 77 | 76 | 85 | 83 |
|  | **observer 4** | **93** | 95 | 95 | 97 | 96 |

**SUPPLEMENTARY VIDEOS**

**Supplementary Video S5: *In vivo* bronchoscopic FCFM of an *Aspergillus fumigatus* infected lung.** The video shows a representative, full image sequence of the lung 3 days post instillation with a GFP-expressing *A. fumigatus* strain. Hyphal structures can be distinguished from the background.

**Supplementary Video S6: *In vivo* bronchoscopic FCFM of a *Cryptococcus gattii* infected lung.** The video shows a representative, full image sequence of the lung 24 days post instillation with a GFP-expressing *C. gattii* strain.

**Supplementary Video S7: *In vivo* bronchoscopic FCFM of a non-infected control lung.** The video shows a representative full image sequence of a healthy, non-infected control lung. No background signals can be detected.

**Supplementary Video S8: Demonstration of the *in vivo* bronchoscopic FCFM procedure.** The animals were fixed in supine position on top of a heating pad to maintain a constant body temperature. To gain easy access to the throat, a small thread was applied behind the front teeth and attached to the heating plate. Subsequently, the tongue was gently pulled sideways and the lower jaw was lifted to obtain a clear view on the tracheal entrance. Next, the inactivated fibre-optic probe (S-300 probe, bevelled or blunt) was gently inserted via the trachea into the lungs. Whenever resistance was felt during insertion, the probe was slightly retracted to prevent puncturing the tissue. FCFM images were acquired while carefully manoeuvring the probe within different parts of both the left and right lungs. The position of the S-300 probe within the lungs could roughly be estimated based on the location of the visible light emitted from the tip of the probe and on the depth of insertion.
